# Supplementary material for: The Effect of Quadruple Therapy with Polaprezinc or Bismuth on Gut Microbiota after Helicobacter pylori Eradication: A Randomized Controlled Trial
Source: J Clin Med. 2022 Nov 29;11(23):7050. doi: 10.3390/jcm11237050 (PMC9739995; doi:10.3390/jcm11237050)
Supplement: Supplementary file 1 [file jcm-11-07050-s001.zip › jcm-2054287-supplementary.pdf]

Supplementary Material

1 Supplementary Data

Inclusion criteria

The enrolled patients should be: 40 to 69 years of age, residents who participated in early screening for upper gastrointestinal cancer and the outpatients at Yanting County Cancer Hospital, with a positive primary ^{14}C urea breath test (UBT) for *H. pylori* infection, with no evidence of gastric cancer or other severe gastric disease identified on gastroscopy, and should provide written informed consent.

Exclusion criteria

Potential participants will be screened on the following exclusion criteria: prior antibiotics or bismuth therapy within 4 weeks of enrolment ; prior PPI therapy within 2 weeks of enrolment; pregnancy or lactation in women; history of cardiovascular and other severe diseases; participation in other clinical studies within 3 months; unable to follow the study procedures (e.g., due to mental illness or severe neurosis); history of allergy to medications used; and any contraindication to gastroscopy.

2 Supplementary Figures and Tables

2.1 Supplementary Tables

Table S1. Baseline demographic and clinical characteristics between the completed and incomplete fecal sample collection populations.

Characteristic	Mean (SD) A set (n=61)	Mean (SD) B set (n=39)	*P*-value
Age (years)	51.20 (4.36)	51.51 (7.14)	0.49
Female gender, NO. (%)	41 (67)	19 (49)	0.066
BMI (kg/m^2)	24.27 (2.55)	24.32 (2.52)	0.88
Education level, NO. (%)			0.74
Primary and below	22 (36)	17 (44)	
Junior	31 (61)	18 (46)	
Senior and above	8 (13)	4 (10)	
Place of residence, NO. (%)			1.00
City	5 (8)	3 (8)	
Countryside	56 (92)	36 (92)	
The source of potable water, NO. (%)			0.070
well water	38 (62)	31 (79)	
tap water	23 (38)	8 (21)	
Active smoking, NO. (%)	10 (16)	14 (36)	0.026
Alcohol consumption, NO. (%)	14 (23)	16 (41)	0.054
Tea consumption, NO. (%)	12 (20)	14 (36)	0.071
The results of gastroscopy, NO. (%)			1.00
Gastritis	60 (98)	39 (100)	
Family history of cancer, NO. (%)	11 (16)	6 (15)	0.73

A set: fecal samples collected

B set: fecal samples uncollected

Table S2. The prevalence rates of gastrointestinal symptoms during treatment [Events/Total (%)]

Symptoms	Day-7		*P*-value	Day-14		*P*-value
	PQT	BQT		PQT	BQT	
Epigastric pain	1/49(2.0)	1/51(2.0)	1.00	2/49(4.1)	1/51(2.0)	0.97
Chest discomfort	1/49(2.0)	-	0.49	-	-	-
Hunger	2/49(4.1)	1/51(2.0)	0.97	2/49(4.1)	1/51(2.0)	0.97
Nausea	4/49(8.2)	-	0.12	4/49(8.2)	1/51(2.0)	0.34
Bloating	4/49(8.2)	4/51(7.8)	1.00	3/49(6.1)	3/51(5.9)	1.00
Sore throat	1/49(2.0)	-	0.49	2/49(4.1)	1/51(2.0)	0.97
Constipation	-	1/51(2.0)	1.00	-	1/51(2.0)	1.00
Diarrhea	-	-	-	-	1/51(2.0)	1.00

2.2 Supplementary Figures

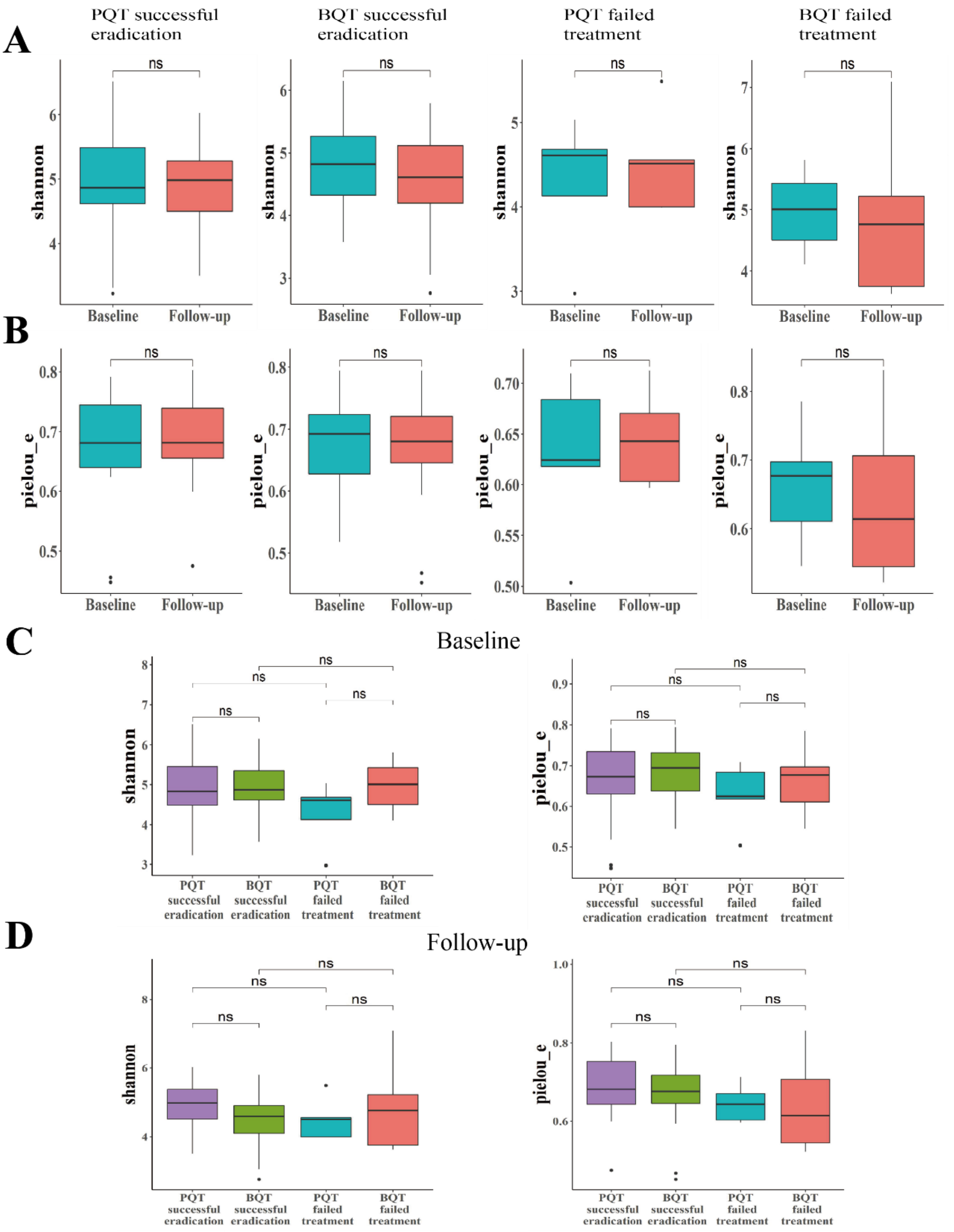

Figure S1. The comparisons of alpha diversities among the four groups. Shannon (A) and pielou evenness (B) indices represent the alpha diversity changes from baseline to the end of treatment. The comparison of intergroup alpha diversities differences at baseline (C) and follow-up (D).

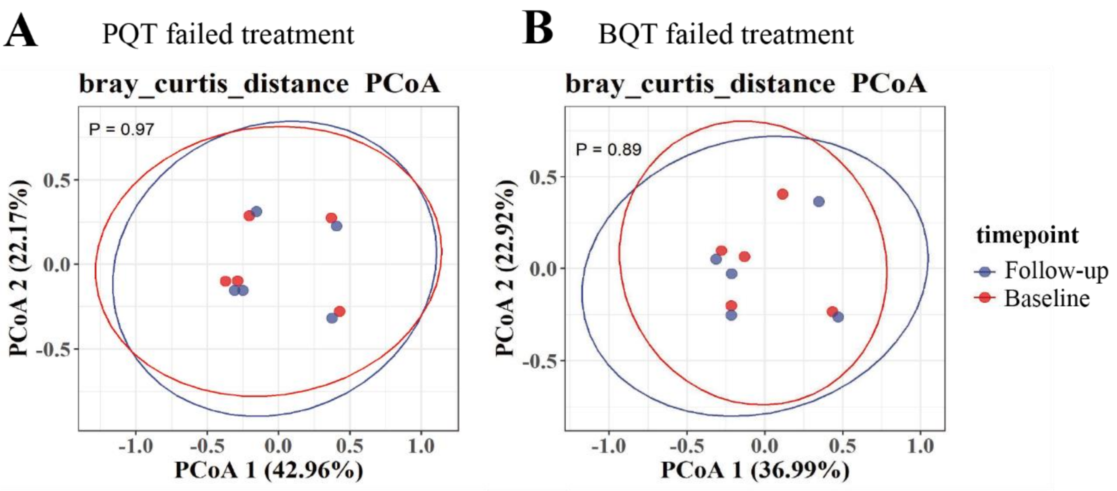

Figure S2. The comparisons of beta diversities. Beta diversity (PCoA) was compared to baseline in PQT failed treatment group (A) and BQT failed treatment group (B).

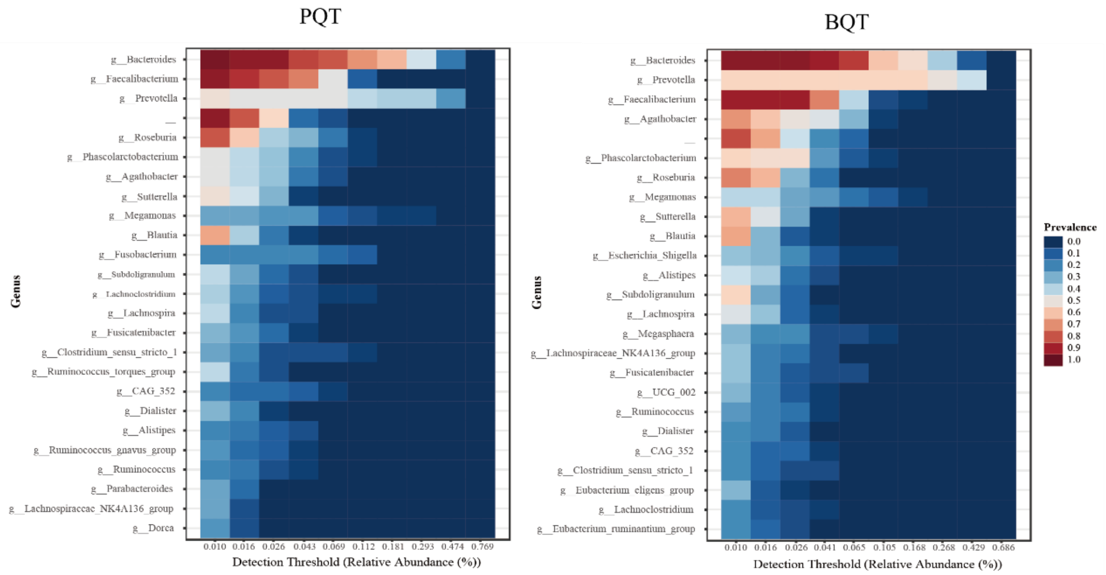

Figure S3. The core microbiota composition between the PQT and BQT at baseline.

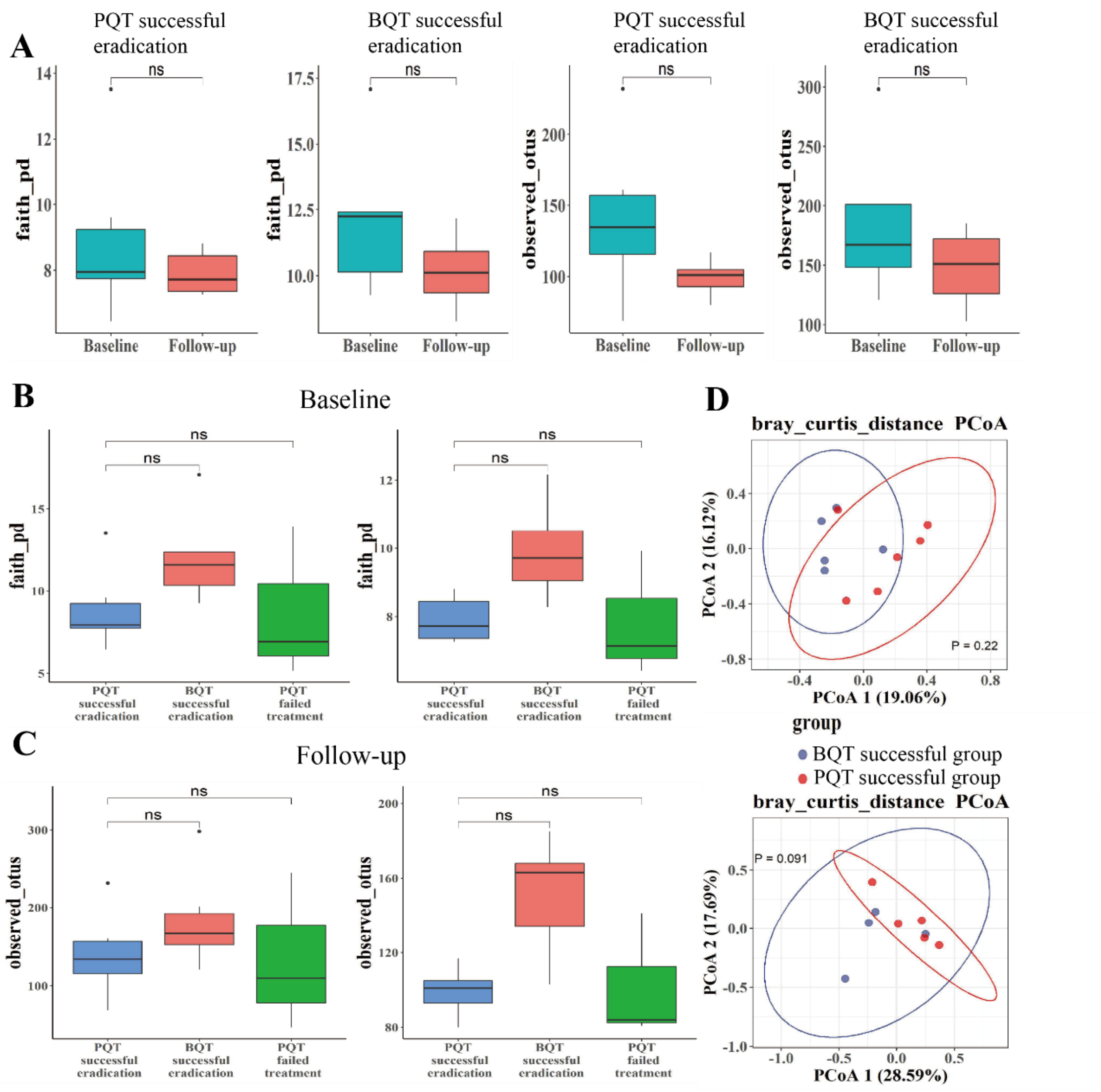

Figure S4. The comparisons of diversities among the three groups that overdue the sample returning. Faith pd and observed otus (A) indices represent the alpha diversity changes from baseline to the day of returning samples. The comparison of between-group alpha diversities differences at baseline (B) and follow-up (C). Beta diversity (PCoA) was compared between the two groups at baseline and follow-up (D).

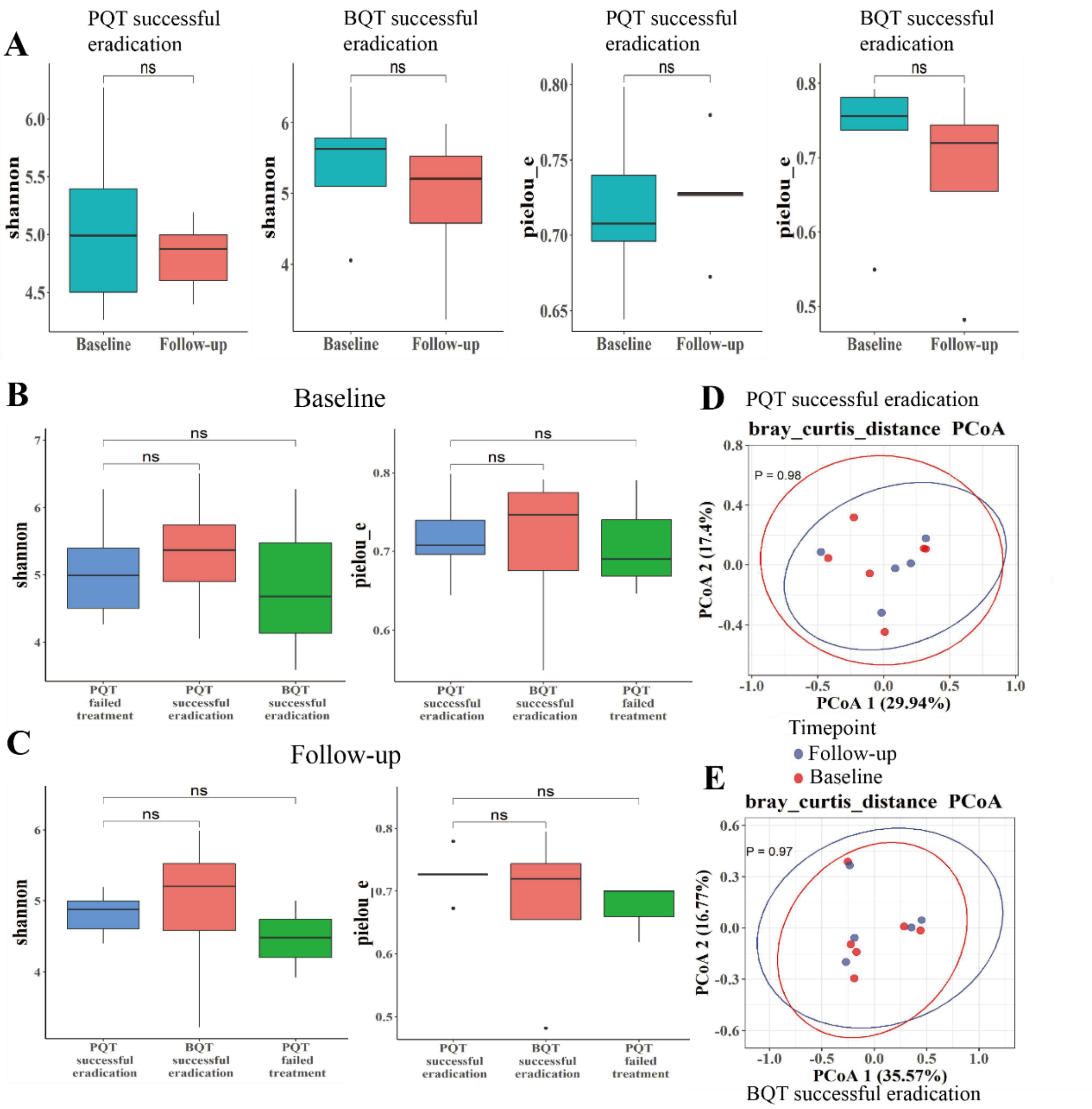

Figure S5. The comparisons of diversities among the three groups that overdue the sample returning. Shannon and pielou evenness (A) indices represent the alpha diversity changes from baseline to the day of returning samples. The comparison of between-group alpha diversities differences at baseline (B) and follow-up (C). Beta diversity (PCoA) was compared at baseline and follow-up in PQT successful eradication group (D) and BQT successful eradication group (E).
